# Comparative Evaluation of Hybrid Attention-CNN and Vision Transformer Models for Multi-Class Classification of Third–Second Molar Relationships on CBCT

**DOI:** 10.3390/diagnostics16132123

**Published:** 2026-07-07

**Authors:** Hazal Karslıoğlu, Jale Bektaş, Lutfiye Sal, Mert Durukan, Mehmet Ozgur Ozemre

**Affiliations:** 1 Department of Oral and Maxillofacial Radiology, Faculty of Dentistry, Mersin University, Mersin 33343, Türkiye; 2 Department of Computer Engineering, Faculty of Engineering, Mersin University, Mersin 33343, Türkiye

**Keywords:** cone-beam computed tomography, deep learning, external root resorption, second molar, third molar, Vision Transformer

## Abstract

**Background/Objectives**: Impacted third molars may adversely affect adjacent second molars, leading to pathological conditions such as external root resorption and dental caries. Accurate assessment of these interactions is important for treatment planning and clinical decision-making. Although cone-beam computed tomography (CBCT) provides detailed three-dimensional imaging, image interpretation remains challenging. Recent advances in artificial intelligence have enabled automated radiographic analysis using deep learning methods. **Methods**: This retrospective study included 162 CBCT scans obtained from patients aged 18–75 years. A total of 306 third molar–second molar units were evaluated. Based on radiographic findings, interactions were categorized as independent, contact, or resorption. Several deep learning architectures were developed and evaluated, including conventional convolutional neural networks (CNNs), attention-based CNNs, and Vision Transformer (ViT) models. Performance was assessed using standard classification metrics, and an ensemble approach was applied to improve predictive stability. **Results**: Attention-based and Transformer-based models generally outperformed conventional CNN architectures. These models achieved better discrimination among the defined classes and demonstrated superior overall performance. The ensemble model produced the most reliable results, achieving the highest macro-area under the curve (macro-AUC) values. Distinguishing contact cases from independent cases was the most challenging task, whereas resorption cases were identified more consistently across different models. **Conclusions**: Transformer-based deep learning models showed promising performance for CBCT-based assessment of third molar–second molar interactions. Ensemble learning further improved classification reliability and robustness. These findings suggest that artificial intelligence-assisted systems may support early detection of third molar-related pathological changes and contribute to more accurate radiological evaluation and clinical decision-making.

## 1. Introduction

Impacted teeth are generally defined as teeth that have completed their development but do not erupt in the dental arch within the expected period or fail to reach a functional position. The prevalence of tooth impaction varies across populations, ranging from 18% to 70%. The most commonly impacted teeth are third molars, accounting for over 95% of all impacted teeth [1]. Because they are usually the last teeth to erupt and root formation occurs later than other teeth, third molars are particularly susceptible to disruptions in their eruption process [2].

The etiology of tooth impaction is multifactorial. A number of contributing factors have been identified, either acting alone or in combination. These include discrepancies between tooth size and jaw dimensions, resulting in arch space deficiency; atypical positioning of the developing tooth; increased density of the overlying soft or hard tissues; ankylosed primary teeth; and the presence of supernumerary teeth that may block the eruption pathway [3].

Impacted teeth are of clinical importance because they may be associated with a range of pathological changes and can influence treatment planning. These pathologies include pericoronitis, dental caries, pain, localized swelling, cysts and tumors, periodontal pathology, and external root resorption (ERR) affecting adjacent teeth [2]. The prevalence of ERR associated with impacted third molars has been reported to range between 20.17% and 81%. Numerous studies in the literature report a strong correlation between distal caries in second molars and impacted third molars. Furthermore, several variables have been identified as potential risk factors for second molars adjacent to impacted third molars, including patient age and sex, the nature of contact between second and third molars, the angulation of the impacted third molar, and the depth of impaction [4,5].

Conventional two-dimensional imaging methods, particularly panoramic radiography, have limitations in detecting early or subtle structural changes due to superimposition and projection geometry. In contrast, cone-beam computed tomography (CBCT) provides three-dimensional visualization, enabling more precise evaluation of anatomical relationships and early pathological changes. In particular, CBCT is recommended for evaluating suspected ERR in second molars, as such defects are often difficult or impossible to detect on conventional two-dimensional images [2]. A comparative study evaluating panoramic radiography and CBCT demonstrated that ERR detection rates were approximately 4.3 times higher with CBCT [2].

In parallel with advances in imaging, artificial intelligence (AI), particularly deep learning, has increasingly been applied in dental radiology. These approaches are capable of handling large and complex datasets with high accuracy and efficiency. In studies involving impacted teeth, such techniques may help reduce the time and effort required for manual evaluation while supporting more consistent and reliable assessments [6].

Most previous studies have focused on AI models based on convolutional neural networks (CNNs) and artificial neural networks (ANNs), which have been widely applied in dental imaging tasks such as the detection of dental caries, apical lesions, and alveolar bone loss, as well as orthodontic assessment [7]. In this context, CNN-based architectures such as ResNet and Mask R-CNN have also been utilized for tasks including third molar localization and periodontal bone loss classification, demonstrating high diagnostic accuracy [8]. Several studies have employed popular CNNs for detection and multiple classification tasks to identify variations around the third molar that correlate with and cause resorption [9,10]. In parallel, deep learning methods for detecting root resorption have been demonstrated on periapical radiographs and CBCT, suggesting feasibility across modalities [11]. Moreover, using Transformer-based models, specifically Vision Transformer (ViT) and Swin Transformer, offers advantages but requires more comprehensive, conceptually grounded information for panoramic radiographs in dental restoration and prosthesis classification [12]. Dental caries detection with an enhanced DenseNet121-C model has achieved accuracies of 93.17% and F1-scores of 87.49% [13]. The global learning capacity of Transformer mechanisms enables hybrid generic CNNs with Transformers, such as TransXNet [14], to generate new models that simultaneously incorporate local and global context. This progression in hybrid models may increase the ability to classify complex structural anomalies. The hybrid architecture of Swin-UNet separates fine structures, such as tooth roots, and can also be adapted in the future to detect similar subtle pathologies, such as resorption [15]. To emphasize the global context learning capacity, another study employed the ViT, YOLO v11, and Faster R-CNN models to detect and classify dental structures, including fillings, crowns, root canal materials, and implants [16]. CNN, Mask R-CNN, and TransUNet models have been used in another study comparing Transformer- and CNN-based classification and detection for diagnosing periodontal bone loss and periodontitis stage, achieving an accuracy of 89.45% [8].

Although deep learning methods have shown promising results in dental imaging, most existing studies are based on two-dimensional images and primarily focus on anatomical classification tasks, such as angulation or impaction depth [7]. There is a lack of studies investigating third molar-related pathological changes, particularly ERR, using CBCT-based multi-class classification approaches. This limitation significantly restricts the clinical applicability of current AI-based systems.

Therefore, this study aimed to evaluate the relationship between impacted third molars and adjacent second molars using CBCT images and to develop a deep learning-based framework for multi-class classification of these interactions. CNN, attention-based CNN, and ViT models were compared under a unified experimental protocol. In addition, an ensemble approach was implemented to improve prediction reliability, and a confidence-based triage strategy was explored to enhance potential clinical usability. To the best of our knowledge, this is the first CBCT-based multi-class classification study of third–second molar interactions using deep learning.

## 2. Materials and Methods

### 2.1. Dataset Acquisition and Eligibility Criteria

This study was approved by the Mersin University Clinical Research Ethics Committee (Approval No: 2026/082; approval date: 4 February 2026) and conducted in accordance with the Declaration of Helsinki. As this was a retrospective archive review, informed consent was waived.

In this retrospective study, CBCT scans obtained between 2024 and 2025 from patients aged 18–75 years who attended the Department of Oral and Maxillofacial Radiology at Mersin University were included in the study. All CBCT images were acquired using the Planmeca ProMax 3D (Planmeca, Helsinki, Finland) with the following parameters: 90 kVp, 5 mA; voxel size: 0.125, 0.25 mm; FOV size: 80 × 80, and 100 × 50 mm. The images were analyzed using the Planmeca ProMax version 4.6.1.R.2017 program on a compatible monitor.

Inclusion criteria were (i) CBCT images demonstrating the presence of both the impacted third molar and the adjacent second molar within the same region, (ii) adequate visualization of the third molar region, and (iii) sufficient image quality for diagnostic evaluation. Exclusion criteria were (i) severe motion or positioning artifacts and (ii) incomplete visualization of the anatomical region.

A total of 162 patients were included in the study. The unit of analysis was defined at the tooth level. Accordingly, 306 third molar–second molar relationships were evaluated. Both left and right sides were evaluated without distinction.

The dataset was divided into training (80%) and test (20%) sets at the patient level. Patients included in the training dataset were not included in the test dataset. Although some patients contributed more than one eligible third molar–second molar region, all image regions from the same patient were assigned exclusively to a single dataset, thereby preventing data leakage.

All CBCT images were systematically reviewed in axial, sagittal, and coronal planes to comprehensively assess the anatomical relationship between the third and second molars. For annotation purposes, sagittal slices were selected, as they provided the most consistent and optimal visualization of the spatial interaction between the two teeth. Image-level classification was therefore performed using representative sagittal slices rather than full volumetric input. This approach was adopted to reduce projection-related ambiguity and ensure labeling consistency across the dataset.

All annotations were performed on CBCT images, which served as the reference standard for classification due to their three-dimensional imaging capability. Image evaluation was conducted by an Oral and Maxillofacial Radiology resident (Lutfiye Sal) and an experienced dentomaxillofacial radiologist (Hazal Karslıoğlu) with over 8 years of experience. To assess inter-observer reliability, 50 randomly selected CBCT images were independently evaluated by both observers. Cohen’s kappa analysis demonstrated substantial agreement (κ = 0.80). In cases of disagreement, a consensus was reached.

### 2.2. Class Definitions and Labeling Protocol

The relationship between third molars and second molars was categorized into three groups based on CBCT findings:

Contact (Class 0): Direct contact between the third molar and the second molar (in contact);

Independent (Class 1): No contact between the third molar and the second molar (independent);

Resorption (Class 2): Contact present, accompanied by ERR in the second molar (resorption).

In addition to the primary classification task, impaction characteristics were evaluated using Winter’s classification [17] for angulation (vertical, mesioangular, distoangular, and horizontal) in both maxillary and mandibular third molars. The vertical component of the Pell and Gregory classification (Classes A–C) was applied to assess the depth of impaction in both jaws [18].

Annotation was performed using LabelMe. Each record was stored as a base64-encoded JPG inside a JSON file; labels were stored in the shapes field as categorical tags (contact/independent/resorption) along with optional point-based annotations. Since the primary objective of this study was image-level classification, class labels were directly used for model training and evaluation. Point annotations were retained to support potential future region-based analyses.

In this study, a dataset of 306 samples was resized to 224 × 224 pixels before training. Augmentation included horizontal flips, with a probability (p) of 0.5, rotation angle to ±15°, and affine translations set to 0.1. For brightness, contrast, and saturation, color jittering was set to 0.2. Augmentation operation resulted in a total of 1835 sample images. Inputs were normalized using ImageNet statistics with mean values of (0.485, 0.456, 0.406), and standard deviation values of (0.229, 0.224, 0.225) [19].

### 2.3. Deep Learning Methods

In this study, a set of deep learning models was employed to perform image-level classification and evaluate multiple architectural families.

The study focuses on widely used CNN-based backbones such as SE-ResNet-50, CBAM-ResNet-50, DenseNet + Attention, and EfficientNet + Attention, along with a baseline CNN and a reference YOLO11 detection trial. Attention modules such as CBAM [20] and squeeze-and-excitation (SE) can recalibrate feature responses and highlight discriminative patterns. Therefore, attention modules were incorporated into deep learning models such as ResNet, DenseNet, and EfficientNet.

Baseline CNN

The baseline CNN provides a lower-bound reference for purely convolutional local feature learning. The model follows stacked convolution blocks, followed by an MLP classifier head and a 3-class softmax output. It was trained as an image-level classifier with the same input resolution, augmentation, and optimizer settings as all other models to ensure comparability.

ResNet-50 with attention modules

ResNet-50 is a deep residual network that enables stable training through residual connections. In this study, two attention-augmented ResNet variants were employed to probe whether attention-based feature re-weighting improves discrimination on CBCT.

SE-ResNet-50

SE-ResNet-50 introduces SE channel gating to learn which feature channels should be emphasized or suppressed [21]. The model was fine-tuned for 3-class image-level classification under the unified protocol. Channel-wise recalibration is relevant for CBCT images because subtle intensity and structure cues may be distributed across channels, and emphasizing informative channels can improve separability.

CBAM-ResNet-50

CBAM-ResNet-50 augments ResNet-50 with channel and spatial attention to capture both the “what” and “where” aspects of discriminative features [20]. The model was trained as a 3-class classifier under the same pipeline to isolate the effect of spatial attention.

DenseNet + Attention

DenseNet employs dense feed-forward connectivity, allowing later layers to reuse feature maps from earlier layers. In this study, an attention re-weighting module was integrated on top of the DenseNet backbone to emphasize informative features before classification, consistent with the general motivation of attention mechanisms. The model was fine-tuned for the 3-class under the same protocol.

EfficientNet + Attention

EfficientNet proposes principled scaling of network depth/width/resolution to improve performance under fixed compute budgets. The EfficientNet + Attention variant was trained as a 3-class CBCT classifier under the same protocol.

YOLO11

A YOLO11 detection trial was included to probe whether a localization-driven paradigm offers benefits over image-level classification. YOLO-style models are standard for efficient detection and are widely used for object localization tasks [22].

However, since the target output of this work is an image-level class label, detection outputs have not been transformed into competitive classification performance and were retained only for contextual comparison.

### 2.4. Transformer Methods

Transformer methods—ViT and Swin Transformer—were evaluated in addition to convolutional architectures [23]. Transformers rely on attention to model contextual relationships across an image. ViT uses patch-based global self-attention, while Swin introduces hierarchical shifted-window attention to support multi-scale representation learning.

Vision Transformer (ViT)

ViT processes images by splitting them into fixed-size patches and applying Transformer encoders with multi-head self-attention. In our experiments, ViT-B/16, ViT-B/32, and ViT-L/16 were trained as 3-class image-level classifiers under the same 224 × 224 input protocol. Global attention may be beneficial for borderline cases where subtle geometric context influences the final label.

Swin Transformer

Swin Transformer employs hierarchical stages with shifted-window attention to support scalable, multi-scale feature learning. In our experiments, Swin-T, Swin-S, and Swin-B were evaluated as 3-class classifiers using the same preprocessing and optimization pipeline.

Swin-S + head-level MHA (Swin-S + MHA(head))

To explore an additional refinement mechanism, we evaluated a Swin-S variant with an extra head-level multi-head attention (MHA) block prior to classification. The architecture can be summarized as Swin-S backbone usage with extra MHA (head-level). This model was used both as a strong single-branch classifier and as the highest-weight component of the ensemble (w_1_ = 1.0). Head-level attention is intended to re-weight high-level features immediately before classification, potentially emphasizing subtle, label-relevant cues.

### 2.5. Proposed Approach: Weighted Soft-Voting Ensemble + Confidence-Based Triage

**Figure 1:**
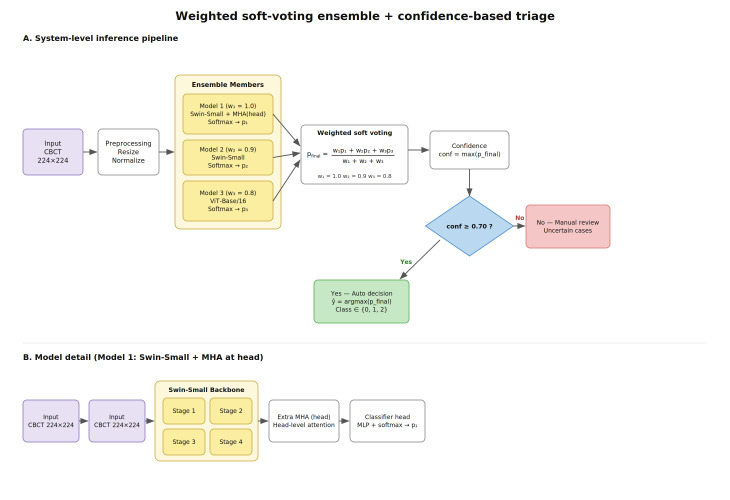
Overview of the proposed tooth resorption classification system. (**A**) Weighted soft-voting ensemble inference pipeline with confidence-based triage (threshold conf ≥ 0.70). (**B**) Architecture of the best-performing Swin Transformer-Small + head-l.

The best-performing approach in this study is a system-level method that combines a weighted soft-voting ensemble [24] with a confidence-based triage rule. Ensemble learning is widely used to improve stability and performance by aggregating complementary decision patterns from multiple models [25]. From a deployment perspective, confidence behavior is as important as overall accuracy. The observed separation of correct versus incorrect predictions by confidence supports a practical two-stage triage: high-confidence cases can be automated to accelerate routine workflows, while low-confidence cases are escalated for clinician review. Correct predictions tended to concentrate at higher confidence values, whereas errors were more frequent at lower confidence bands, motivating a two-stage triage strategy. The prediction confidence was defined as the maximum class probability. A two-stage decision rule was applied as follows: if conf ≥ 0.70, an automated label is returned; otherwise, the case is routed to manual review. In the configuration of this study, the ensemble combines Swin-S + MHA(head), Swin-S, and ViT-B/16 with weights w_1_ = 1.0, w_2_ = 0.9, and w_3_ = 0.8, respectively, as shown in Figure 1. The final ensemble probability is computed as Equation (1):

(1) $$p_{final}=\left(\sum_{i}w_{i}p_{i}\right)\left(\sum_{i}w_{i}\right)$$

where w_i_ denotes the weight of each model and p_i_ denotes the probability of each model. Prediction confidence is defined as Equation (2):

(2) $$Conf=max(p_{final})$$

## 3. Results

The distribution of impaction characteristics revealed that the majority of third molars were classified as Pell and Gregory Class C (*n* = 124). Regarding angulation, vertical (*n* = 81) and mesioangular (*n* = 65) positions were the most commonly observed patterns.

The dataset included 306 third molar–second molar units, with the class distribution as follows: Contact (*n* = 130, 42.5%), Independent (*n* = 84, 27.5%), and Resorption (*n* = 92, 30.0%).

Validation preprocessing was applied only at the resizing, tensor conversion, and normalization stages. To standardize third molar-related screening on CBCT images as a three-class problem, CNN-, Transformer-, and attention-based models were evaluated for Contact/Independent/Resorption classification under a unified experimental protocol, followed by an ensemble strategy. For model performance evaluation, a stratified hold-out validation strategy was employed to preserve class distributions, using an 80% training subset and a 20% validation subset; the random seed was set to 42. To balance the results, random seeds for NumPy, scikit-learn, and PyTorch 2.9.1 libraries were fixed; however, deterministic computation was not enforced, and PyTorch’s non-deterministic behavior was allowed. During all preprocessing steps, such as resizing, tensor conversion, and normalization, model training was performed, estimating scaling parameters exclusively on the training set. To prevent information leakage, the validation set was used only for performance evaluation. Aggregated analyses, such as full confusion matrices and confidence threshold sweeps, were reported using predictions generated on the validation dataset for descriptive purposes. Analysis results were interpreted with the most appropriate scope hold-out validation versus full-dataset descriptions.

### Evaluation Criteria

Several metrics were used for performance evaluation, including accuracy, macro-precision, macro-recall, macro-F1, and macro-AUC (OvR). These metrics provide valuable insights into the model’s ability to make accurate predictions on unclear class predictions and are widely employed in model selection and performance comparison. Additionally, a weighted linear composite score metric [26] to rank the benchmarking models was computed for evaluating macro metrics together. Composite score values were calculated using Equations (3) and (4), respectively.

(3) $$Composite=w_{1}M_{1}+w_{2}M_{2}+\ldots +w_{n}M_{n}$$

(4) Score=0.444Macro−F1+0.333 Macro AUC+0.222Accurcy

The overall performance across methods is summarized in Table 1. The weighted soft-voting ensemble achieved the best results, with an accuracy of 96.73%, a macro-F1 of 96.80%, a macro-AUC of 99.71%, and a composite of 97.66%. Among benchmark models, the highest composite scores clustered around ~0.90: swin_small (92.70), vit_base_16 (92.67), and swin_tiny (92.46). The baseline CNN and the YOLOv11 trial performed substantially worse, with composite values ranging from 0.35 to 0.37.

**Figure 2:**
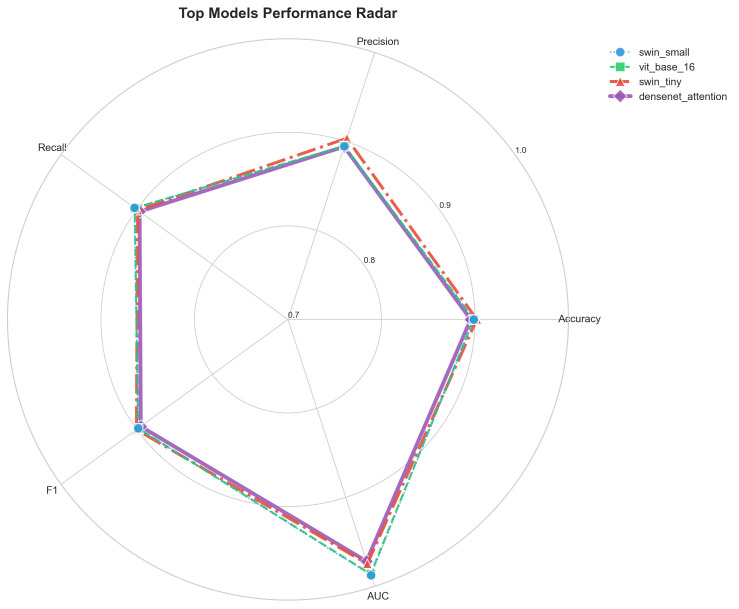
Performance radar graph of the four methods showing by far the best performance, indicating precision, recall, F1, AUC, and accuracy evaluation metrics.

To visualize family-level differences, accuracy, macro-F1, and macro-AUC results were compared across representative models (Figure 1). Metric profiles of the top single models were incorporated using a radar plot (Figure 2).

**Figure 3:**
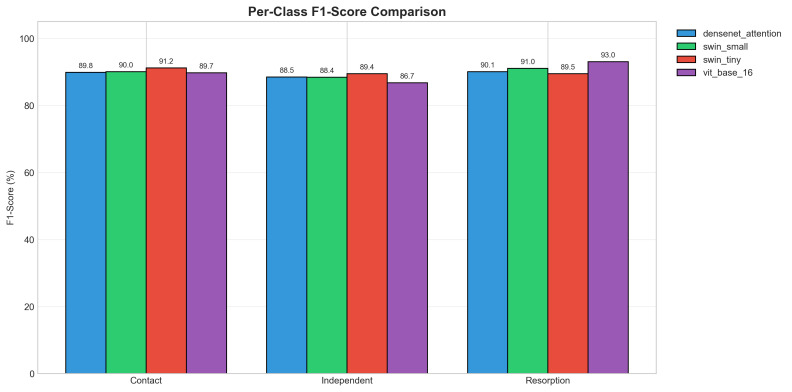
Per-class F1-score comparison. Class-wise F1 for Contact, Independent and Resorption across selected models.

**Figure 4:**
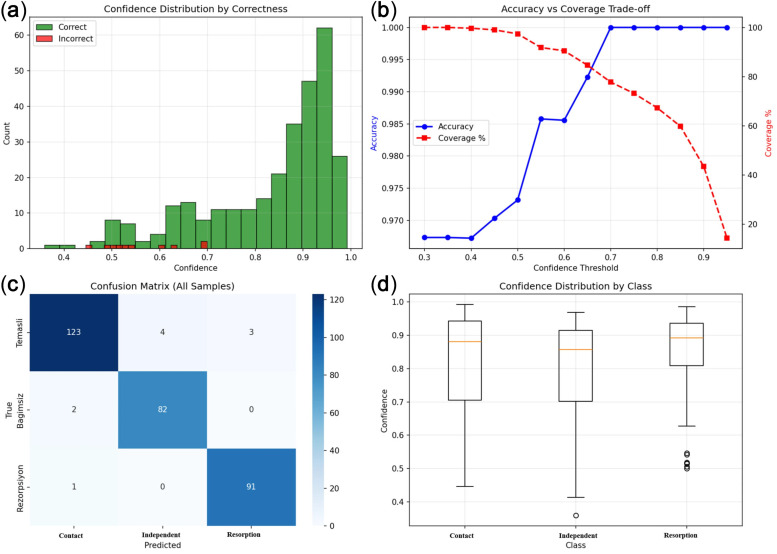
Confidence analysis (all samples). (**a**) Confidence distributions for correct/incorrect predictions, (**b**) accuracy–coverage trade-off, (**c**) confusion matrix (all samples), and (**d**) confidence distribution by class.

Regarding class-wise performance and error patterns, the distribution of F1-scores across classes is shown in Figure 3. Overall, stable performance was observed across all three classes; however, Resorption class samples tended to benefit more from Transformer-based representations, particularly ViT-based variants. Normalized confusion matrices for the top three single models are presented in Figure 4, suggesting that most errors concentrate along the Contact vs. Independent axis, while Resorption is comparatively stable. Class-wise results and confusion patterns indicate that the most challenging distinction lies between Contact and Independent cases, where subtle positional cues may be affected by projection geometry and superimposition. In contrast, the Resorption class appears more stable across top models, and ViT-based variants show a partial advantage, consistent with the hypothesis that global context and longer-range dependencies can aid in recognizing localized but context-dependent cases.

**Figure 5:**
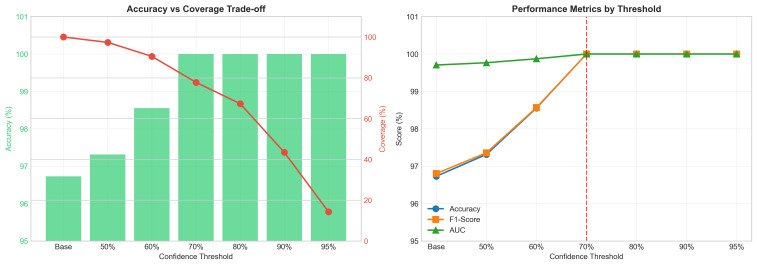
Ensemble threshold analysis. (**left**) Accuracy–coverage trade-off; (**right**) accuracy, F1, and AUC across thresholds (highlighted: 70%).

Regarding confidence-based clinical triage, the ensemble model’s confidence behavior was examined (Figure 4). Confidence was defined as an accuracy–coverage trade-off, which was evaluated by sweeping confidence thresholds. At threshold 0.0, the ensemble achieved 96.73% accuracy with 100% coverage. Increasing the threshold reduced coverage while improving reliability on the retained subset. Test-time augmentation (TTA; 7 transforms) was evaluated for Swin-S + MHA(head) and yielded an overall accuracy of 80.07%. These findings support a two-stage workflow in which high-confidence cases were automatically labeled, and low-confidence cases are routed to manual review. Threshold sweeping (Figure 5) indicated that at 0.70, automated decisions covered ~77.8% of cases while preserving error-free performance in the high-confidence subset reported in the descriptive analysis.

Explainability results using input-gradient saliency maps suggested that decisions are driven by anatomically plausible tooth and root regions [27]; complementary methods such as Grad-CAM may provide additional insight [28].

**Figure 6:**
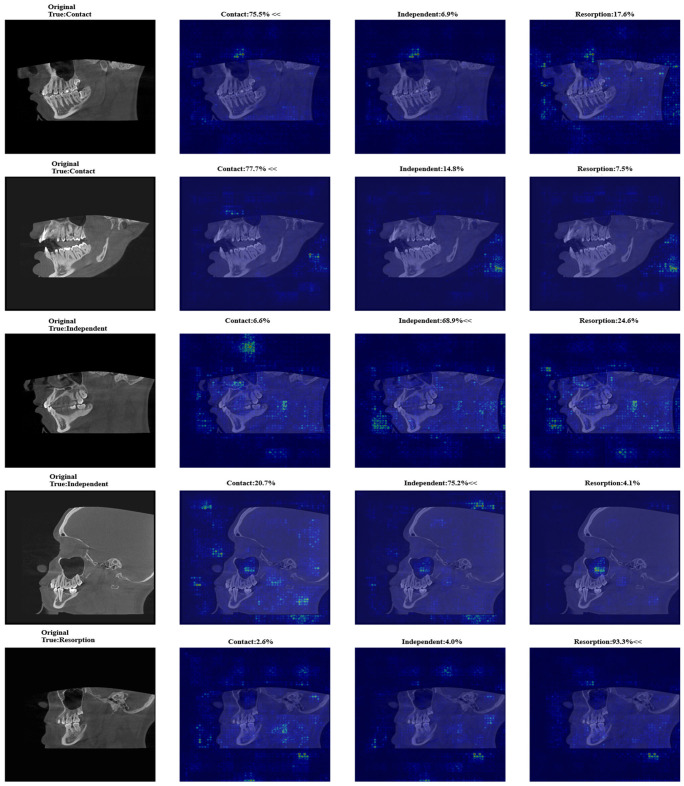
Saliency examples. Class-wise saliency maps with predicted probabilities on representative cases.

Uncertainty estimation using MC Dropout did not provide discriminative epistemic uncertainty in this configuration. Uncertainty values were near zero in practice and did not distinguish between correct and incorrect predictions, suggesting limited utility. In contrast, input-gradient saliency maps (Figure 6) indicated that model attention was concentrated in clinically plausible tooth and root regions.

## 4. Discussion

Unlike previous studies primarily based on two-dimensional imaging and binary classification, the present study introduces a CBCT-based multi-class framework combined with a confidence-driven triage strategy, potentially improving both diagnostic precision and clinical workflow integration. In this study, Transformer- and attention-based models outperformed conventional CNN architectures in classifying third molar-related interactions on CBCT images. The ensemble approach achieved the highest performance, indicating improved stability and class discrimination. In clinical practice, this approach could help identify third molar-related pathologies at an earlier stage and support treatment planning, particularly by enabling clinicians to recognize higher-risk cases that may require closer follow-up.

In dental radiology, deep learning algorithms have been increasingly applied for image detection, segmentation, and classification tasks, supporting the diagnosis of conditions such as dental caries, cysts, and maxillofacial lesions. Yılmaz et al. [29] employed a YOLOv4-based architecture for automated tooth classification on panoramic radiographs, while Zirek et al. [30] proposed a model capable of detecting impacted teeth and categorizing them according to the Winter classification system using panoramic imaging.

Vinayahalingam et al. [31] trained MobileNet V2 to distinguish carious third molars from non-carious cases on panoramic radiographs and achieved an accuracy of 87% and an AUC of 0.90 for this classification task. Similarly, object detection approaches such as Faster R-CNN and YOLO-based models have demonstrated high performance in detecting impacted third molars on panoramic radiographs.

Recent studies have further demonstrated the expanding role of deep learning in dental radiology, particularly in the automated classification of impacted third molars. A YOLOv11-based model demonstrated a high accuracy rate in classifying impacted third molars according to the Pell and Gregory vs. Winter classification. These findings suggest that AI can be used to standardize complex radiographic interpretations and reduce observer variability [6].

In addition to radiographic detection studies, the literature also includes studies examining the clinical impact of impacted third molars on adjacent second molars. In a study involving 2642 mandibular second molars, machine learning models achieved high predictive performance (AUCs of 0.88–0.89) for identifying distal caries risk associated with impacted third molars. These findings highlight that third molar–second molar interactions are not limited to structural changes such as resorption, but also include clinically significant outcomes such as caries development, further emphasizing the importance of early and accurate assessment [5]. In another panoramic study, a YOLOv8-based model demonstrated high performance in multi-class classification of caries and ERR, while also improving clinicians’ diagnostic sensitivity and reducing interpretation time. These findings highlight the clinical potential of automated systems in supporting decision-making [32].

While these studies provide valuable insights into automated detection in panoramic radiographs, they offer limited information due to the imaging method’s two-dimensional nature; small pathological findings may not be visible in two-dimensional imaging. In contrast, the use of CBCT in the present study enabled three-dimensional evaluation without magnification or superimposition, thereby enabling more precise assessment of anatomical relationships and early-stage resorptive changes [33]. This difference in imaging method in our study may partially explain the improved discriminative performance observed in our models. Accordingly, the classification task was formulated as a three-class problem (Contact, Independent, and Resorption), allowing for a more comprehensive evaluation of the relationships between the third and second molars.

Class-wise results and confusion patterns indicate that the most challenging distinction lies between Contact and Independent cases, where subtle positional cues may be affected by projection geometry and superimposition. In contrast, the Resorption class appears more stable across top models, and ViT-based variants show a slight advantage, consistent with the hypothesis that global context and longer-range dependencies can aid in recognizing localized but context-dependent signals.

From a deployment perspective, confidence behavior is as important as overall accuracy. The observed separation of correct vs. incorrect predictions by confidence supports a practical two-stage triage: high-confidence cases can be automated to accelerate routine workflows, while low-confidence cases are escalated for clinician review. Because confidence values may be miscalibrated in modern neural networks, explicit calibration (e.g., temperature scaling) may further improve decision reliability.

Uncertainty estimation via MC Dropout did not provide discriminative epistemic uncertainty in this configuration. In future work, alternative uncertainty methods, such as deep ensembles, could be considered. Explainability results using input-gradient saliency maps suggested that decisions are driven by anatomically plausible regions (tooth and root); complementary methods such as Grad-CAM may provide additional insight.

Several limitations should be noted. First, the relatively small sample size and single-center design may limit the generalizability of the findings across different populations, imaging protocols, and CBCT devices. Second, some aggregated analyses were performed on full-dataset predictions for descriptive purposes; this is useful for understanding model behavior but should not be conflated with hold-out generalization. Third, the large drop in performance suggests that training stability and protocol design are critical under small-data constraints; longer training and fold-wise tuning may be required. Finally, although annotations include point-based information, the current pipeline focuses on image-level classification; ROI localization/segmentation could improve interpretability and potentially performance.

## 5. Conclusions

Deep learning models can be effectively used in CBCT images to detect the relationship between impacted third molars and second molars, and resorption in second molars associated with third molars. Models incorporating Transformer and attention mechanisms demonstrated more consistent performance, and using an ensemble approach further improved reliability. From a clinical perspective, such systems may support early detection and help clinicians prioritize cases that require closer evaluation. However, validation on larger and multi-center datasets is still needed to confirm generalizability and strengthen clinical applicability.

## Figures and Tables

**Table 1:** Model performance summary: The results of the experimental studies carried out in the study.

Model	Accuracy (%)	Precision (%)	Recall (%)	Macro-F1 (%)	Macro-AUC (%)	Composite
Soft weighted Ensemble Swin-S + MHA(head) + Swin-S + ViT-B/16)	96.73	96.80	96.80	96.80	99.71	97.6566
swin_small	89.87	89.47	90.29	89.81	98.74	92.7072
vit_base_16	89.87	89.49	90.26	89.81	98.62	92.6672
swin_tiny	90.20	90.36	89.88	90.03	97.47	92.4552
densenet_attention	89.54	89.42	89.61	89.46	97.18	91.9590
resnet50_se	88.24	87.83	88.27	88.00	97.78	91.2220
swin_base	85.95	85.90	87.18	85.96	96.93	89.5248
vit_base_32	85.95	86.45	85.07	85.59	94.28	88.4781
efficientnet_attention	85.29	85.58	84.56	84.81	92.79	87.4890
vit_large_16	72.22	81.90	68.32	70.61	88.76	76.9407
resnet50_cbam	43.14	37.33	35.22	26.14	47.79	37.0973
YOLOv11_Detection	42.81	47.54	33.73	20.71	51.71	35.9184

## Data Availability

The original contributions presented in this study are included in the article. Further inquiries can be directed to the corresponding author.

## Abbreviations

CBCT	Cone-beam computed tomography
AI	Artificial intelligence
ERR	External root resorption
